# Implementation of the national community health policy in Guinea: a decision space analysis of the roles and responsibilities of community health workers

**DOI:** 10.1007/s00103-025-04076-8

**Published:** 2025-06-20

**Authors:** Alexandre Delamou, Fassou Mathias Grovogui, Facely Camara, Delphin Kolié, Tohaninzé Goumou, Lior Miller, Amy Nye, Thomas Bossert

**Affiliations:** 1 Centre National de Formation et de Recherche en Santé Rurale (CNFRSR), Maférinyah, Guinea; 2 Africa Center of Excellence (CEA-PCMT), Gamal Abdel Nasser University, Conakry, Guinea; 3 Ministry of Health and Public Hygiene, Conakry, Guinea; 4 Health Systems Strengthening Accelerator, Results for Development, Washington, United States; 5 Department of Global Health and Population, Harvard T.H. Chan School of Public Health, Massachusetts, United States

**Keywords:** Community health policy, Decentralization, Decision space, Implementation science, Guinea

## Abstract

**Introduction:**

Community health workers (CHW) are crucial for universal health coverage (UHC) in low- and middle-income countries. Decentralization supports this goal but can cause issues if local actors misunderstand their roles. This study explores how Guinea’s local health system actors understood and executed their responsibilities in delivering community health services from 2017 to 2021.

**Methods:**

This is a subanalysis of 168 CHW from a larger study of 522 respondents on the implementation of community health policy in Guinea. We used a sequential explanatory mixed-methods design to assess the knowledge, involvement, and decision space of national community health policy actors, focusing on CHW and community volunteers (*Relais communautaires* [RECO]). This analysis was guided by decision space theory and explored both de jure (formal) and de facto (actual) decision-making. De jure decision space refers to the choices authorized by official strategies, policies, or laws, while de facto decision space reflects the choices reported by local actors in practice.

**Results:**

Across all commune types, CHW and RECO demonstrated high knowledge and implementation levels of their roles in community health. Contrary to the initial hypothesis that fully implemented communes would have the highest decision-making space, commune type was not the primary determinant of decision-making space for CHW and RECO. The presence of CHW was positively associated with greater de jure and de facto decision-making space and enhanced capacity (*p* = 0.050). Univariate analysis showed that increased BCG vaccination coverage (vaccine to protect against tuberculosis) was significantly associated with expanded de jure decision-making space (*p* = 0.041). Bivariate (*p* = 0.014) and multivariate (*p* = 0.011) analyses revealed that higher pentavalent vaccination coverage (vaccine to protect against diphtheria, tetanus, pertussis, hepatitis B, and *Haemophilus influenzae* type b) was also significantly associated with a larger de jure decision-making space.

**Conclusion:**

In countries with decentralized responsibilities, it is crucial for community health actors to understand their decision-making range to improve health outcomes. Also, ensuring sufficient and consistent capacity and funding is essential to enhance health services.

**Supplementary Information:**

The online version of this article (10.1007/s00103-025-04076-8) contains supplementary material, which is available to authorized users.

## Introduction

Community health workers (CHWs) play an essential role in providing preventive and curative primary care, contributing to the achievement of universal health coverage and improved health outcomes, particularly in low- and middle-income countries (LMICs) [1–4]. Moreover, CHWs and other cadres of lay workers in the health sector [5] are also crucial for enhancing health system resilience, given their key roles in community mobilization, service provision, and community-based surveillance in the context of natural disasters, conflicts, and epidemics [5–7]. Furthermore, CHWs help address the growing shortage of health care workers in LMICs, particularly in rural areas, a significant barrier to attaining global health security and universal health coverage [8].

To enhance the responsiveness and effectiveness of community health systems, many countries have adopted decentralization as a governance strategy, transferring authority and decision-making responsibilities from central to local levels [9]. This approach aims to empower local actors to make context-specific decisions, foster accountability, improve resource allocation, and enable innovative service delivery. However, decentralization is a complex, long-term intervention that involves various stakeholders, including those outside the health sector, who may have divergent interests and influences [10, 11].

Evidence from settings such as Kenya, Indonesia, and South Africa suggests that decentralization can lead to unintended consequences. For instance, preexisting patronage systems may result in the unequal distribution of resources and the prioritization of development programs perceived to contribute more to economic growth over health and social development initiatives [12, 13].

The success of decentralization efforts, therefore, relies on more than just formal policy provisions. Local actors’ ability to understand and fulfill their prescribed roles is often hindered by challenges such as capacity gaps, resource limitations, and persistent centralized control. These issues are particularly well-documented in LMICs, where disparities in access to care and weak accountability mechanisms continue to undermine health system performance [14–16].

Guinea’s national community health policy (NCHP), introduced in 2017, represents a significant step toward decentralized health governance in the country [17, 18]. The NCHP seeks to improve the quality and accessibility of community health services, aligning with broader decentralization reforms [17, 18]. These reforms, formalized through the 2017 Local Authorities Code (Law AN 017), delegated up to 14 key responsibilities to communes, including managing and delivering community health services [18]. By June 2022, the NCHP had been implemented in 75% of Guinea’s communes (257 out of 342) through two distinct models: (i) convergent communes or fully implemented communes, integrating NCHP activities into local development plans with partner funding, and (ii) operational communes or partially implemented communes, where community health strategies are supported independently by donor-funded implementation partners.

Despite these efforts, significant challenges persist. Overlapping roles, resource limitations, and deviations from prescribed responsibilities have raised questions about the policy’s effective implementation. Initial evidence (unpublished report) from the evaluation of the Community Health Strategy in Kindia and Telimele in 2021 in Guinea suggests that local actors often struggle to navigate their new roles within the decentralized framework, resulting in misaligned practices and unfulfilled policy objectives. These gaps highlight the need for a deeper understanding of how decentralized actors interpret and execute their roles in delivering community health services.

This study examines the extent to which community health actors (CHW and community volunteers [*Relais communautaires*, or RECO]) in Guinea were aware of, understood, and fulfilled their roles and responsibilities under the NCHP between 2017 and 2021. Using the “decision space” framework, the study explores the alignment between policy intentions, including the de jure decision space (the degree of choice that local actors are authorized to make as is written in official strategies, policies, or laws) and on-the-ground practices, including the de facto decision space (the degree of choice that local actors report that they exercise), providing critical insights into the challenges and opportunities of decentralized community health governance [19]. The findings aim to inform strategies to strengthen health systems in Guinea and other LMICs, advancing efforts toward universal health coverage and global health equity.

## Methods

### Study setting

The study was conducted in Guinea, a country located in West Africa that has approximatively 15 million inhabitants. Most of the population is illiterate (55% in 2021) [20] and lives in rural areas (63%) [21]. Approximately 44% of the population lives below the national poverty line [17]. The country is subdivided into seven administrative regions, comprising 33 prefectures and the capital, Conakry, which includes nine urban communes [21]. In Guinea, maternal and child health coverage remains suboptimal: Only 35% of pregnant women received at least four antenatal care visits in 2018, and 56% gave birth in a health facility. Additionally, only 24% of children aged 11 to 23 months are fully immunized. The health system is organized in a pyramid from national to community level. The health districts cover a prefecture and include a number of subdistricts (also known as communes), which usually host a health center. The implementation of the NCHP started in 2018 as a pilot program in 40 communes and has since been extended to 257 communes. The objective of the policy is to cover all communes across the country’s eight administrative regions and four natural regions. The plan is for a total of 18,938 RECO and 1895 CHW to be recruited and deployed in 338 of 365 total communes in Guinea.

This implementation research covered all categories of communes and regions to ensure a broad and in-depth understanding of the NCHP. After discussion with the National Directorate of Community Health and Traditional Medicine, the following four study regions were purposely selected to reflect variation in the status of NCHP implementation: Kindia, Mamou, Labé, and N’Zérékoré. Within these regions, we included (1) the fully implemented communes (according to the criteria of the Ministry of Health), representing 40 communes that had benefited from the transfer of 14 competencies, including in health, education, and public health, through the National Program of Support for Convergent Communes and Community Health and the decentralization process of the revised code of local governments; (2) the partially implemented communes, representing communes where the NCHP was fully implemented according to Ministry of Health criteria, with the support of development partners but where the official Ministry decentralization process and policies had not yet been rolled out; and (3) the control communes, including communes that had not yet been targeted for rollout of the policy or where CHW/RECO were initiated but not yet fully operational.

### Theoretical framework

This analysis was guided by the decision space framework, originally developed by Bossert et al. [20], to evaluate decentralization reforms in health systems of LMICs [19]. The decision space approach defines decentralization as the range of choice over different functions that subnational actors exercise as formally defined by their roles and responsibilities (de jure decision space) and what they actually decide in practice (de facto decision space). The approach also analyzes the capacities of the actors in terms of their skills in making good decisions and how higher authorities and the communities hold them accountable for their decisions. The framework stipulates that the decentralization and health reforms in Guinea, including the NCHP, the National Decentralization Policy, and the Local Development Plan, would improve community health in Guinea, including health outcomes and community satisfaction with services offered by CHW and RECO because of these new policies and strategies. These policies and strategies were expected to lead to better community health outcomes and improved performance of community health programs in a decentralized setting [19] by improving the decision space, capacities, and accountability in the community health system [22].

It was expected that these improvements would be observed and measured by improved knowledge and practice of de jure and de facto decision space, increased financial and organizational capacities, increased qualified and trained human resources, increased knowledge and capacities of stakeholders, and improved accountability.

### Study design

The design of the larger study on implementation research used a realist evaluation approach [23] based on a sequential explanatory model with mixed methods, including the use of quantitative and qualitative methods [24]. We developed a survey to measure decision space, capacity, and accountability concepts as well as community satisfaction with health services. We analyzed routine health service data from health facilities and the District Health Information System-II (DHIS2). The realistic evaluation approach was used because of the complexity of the NCHP, involving multiple actors and stakeholders across administrative levels. This approach aligned with the study’s theory of change, enabling the exploration of how contextual factors such as governance and resources interact with mechanisms such as decision space and institutional capacity to produce observed outcomes. The sequential mixed-methods design—quantitative followed by qualitative—further supported the realist objective of explanatory depth by first identifying patterns and then unpacking their underlying drivers (the full study protocol has been published elsewhere [23]).

The current subanalysis focuses on analyzing the de jure and de facto decision space among CHW and RECO, including their knowledge and implementation of their responsibilities of the NCHP and related decentralization policies, capacities to implement the NCHP, and accountability in the three types of communes.

The typology of communes (fully implemented vs. partially implemented vs. control) reflects varying degrees of decentralization and external support. This classification allows us to assess how decentralized authority and implementation support influence local actor performance and decision space. Fully implemented communes are the desired model by the Ministry of Health, since in this model, community health and decentralization are essentially managed by local stakeholders and fully funded by local development budgets.

### Study population and data

The study was conducted among stakeholders involved in implementing the NCHP at various levels, from the national to the commune level. Out of Guinea’s 343 total rural communes, the study sample included six fully implemented communes, nine partially implemented communes where the NCHP was functional, and 12 control communes where the NCHP was not functional. Additional details on the sampling strategy are available in the study protocol [23]. The target population of the larger study included 522 respondents, of whom 168 from the community level were considered for this subanalysis. Data were collected between January and February 2022 by 12 trained data collectors through face-to-face interviews using KoboToolbox [25].

### Data analysis

Data were analyzed using Stata software (version 17). First, we described actors’ knowledge of the NCHP, roles and responsibilities, decision space, and accountability using proportions (with 95% confidence intervals) and means (with standard deviation). Except for sample characteristics, results were stratified by type of commune.

Second, based on existing literature and theory [19, 22, 26], we constructed indexes for de jure decision space, de facto decision space, accountability, and capacity. These indexes were created using summative scores derived from variables related to actors’ policy knowledge, assigned roles and responsibilities, organizational and financial capacities, and accountability mechanisms. The de jure index captured knowledge of roles and responsibilities using 13 items reflecting formal tasks. The de facto index measured implementation using 34 items related to field-level activities. The capacity index included 16 items assessing resource availability, training, and financial support. The accountability index comprised eight items related to performance monitoring and reporting mechanisms. For all indexes, each item was coded as 0 (“no”) or 1 (“yes”) and then summed [24].

To compare mean index scores across the three commune types, we used one-way analysis of variance (ANOVA). Prior to conducting ANOVA, we assessed the homogeneity of variances using Bartlett’s test. Nonsignificant *p*-values (> 0.05) indicated that the assumption of equal variances was met, supporting the use of ANOVA.

Finally, we applied ordinary least squares (OLS) regression to explore associations between commune type and each of the four indexes (de jure decision space, de facto decision space, capacity, and accountability). Covariates included the proportion of the rural population, the proportion of the population living below the national poverty line, the number of confirmed malaria cases, and the coverage of key maternal and child health services including BCG vaccine (to protect against tuberculosis) and penta or pentavalent vaccine (to protect against diphtheria, tetanus, pertussis, hepatitis B, and *Haemophilus influenzae* type b).

All statistical analyses were performed at the 5% level, and differences in levels and trends were considered statistically significant at a value of *p* < 0.05.

### Ethical considerations

In this study, all in-depth interviews and focus group discussions took place in private locations. Names were not associated with notes or other study materials. Free and informed consent was obtained verbally from the participants (given the context of the Ebola virus disease at the time of data collection). However, the process was documented electronically on data-collection tablets. All recordings made during interviews or photos taken for the purposes of the study required a completed consent form.

## Results

### Characteristics of study participants

Overall, 168 CHW/RECO participated in the survey. Of these, 51 (30.4%) were from Nzérékoré region, and 46 (27.4%) were from Kindia region. The CHW/RECO had a mean age of 36.1 years, and most of them (115; 68.5%) were male. The majority (94%) were above the primary level of education, and only about 15% of CHW/RECO had training from a health school.

Among CHW/RECO, 26.1% were from fully implemented communes, 32.2% were from partially implemented communes, and 41.7% were from control communes (Table 1).

**Table 1:** Sociodemographic characteristics of community health workers and community volunteers interviewed, Guinea, February 2022

Characteristics	N	% [95% CI]
*Region*		
Kindia	46	27.4% [95% CI: 21.1–34.7]
Labé	27	16.1% [95% CI: 11.2–22.5]
Mamou	44	26.2% [95% CI: 20.1–33.4]
N’Zérékoré	51	30.4% [95% CI: 23.8–37.8]
*Mean age (standard deviation)*	–	36.1 (11.0)
*Gender*		
Female	53	31.6% [95% CI: 24.9–39]
Male	115	68.5% [95% CI: 61–75.1]
*Level of education*		
Primary school	27	16.1% [95% CI: 11.2–22.5]
Secondary school	95	56.5% [95% CI: 48.4–64.6]
University	12	7.1% [95% CI: 4.1–12.2]
Primary health school (nursing and midwifery)	2	1.2% [95% CI: 0.3–4.7]
Secondary health school	23	13.7% [95% CI: 9.2–19.8]
Vocational school	14	8.3% [95% CI: 5–13.6]
*Distance from the supervisory health center*		
Less than 30 min	68	40.5% [95% CI: 33.3–48.1]
30 min to less than an hour	42	25% [95% CI: 19–32.1]
1 to less than 2 h	38	22.6% [95% CI: 16.9–29.6]
2 h or more	20	11.9% [95% CI: 7.8–17.8]
*Main mode of transport*		
Walking	86	51.2% [95% CI: 43.6–58.7]
Motorcycle	79	47% [95% CI: 39.5–54.6]
Stroller/bike	1	0.6% [95% CI: 0.1–4.2]
Public transport (bus/car)	2	1.2% [95% CI: 0.3–4.7]
Complete 15-day training on the care package	136	81.5% [95% CI: 74.9–86.8]
Took a refresher training	88	52.4% [95% CI: 44.8–59.9]

### Knowledge, capacities, and implementation of NCHP responsibilities

**Fig. 1:**
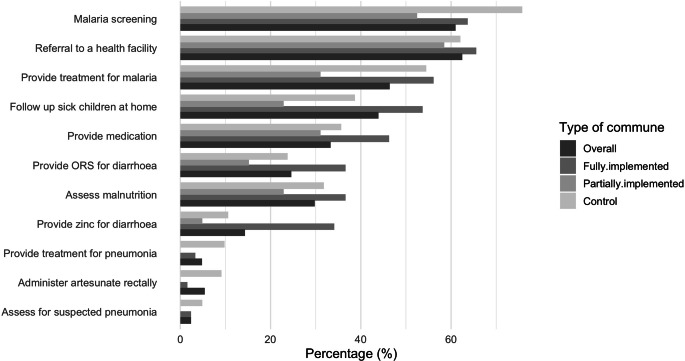
Distribution of community health workers and community volunteers according to the various curative services offered by type of commune, Guinea, February 2022. *ORS* oral rehydration solution

Table 2 presents the mean index of the four dimensions of decision space. The CHW/RECO had a high level of knowledge of their roles in implementing the NCHP. The average level of knowledge was 11.90 (SD = 1.7) out of a total index of 13 (de jure index—knowledge of assigned roles; Table 2). That average was statistically similar across all three types of communes (*p* = 0.863). For example, at least 98% of CHW and RECO in all three types of communes reported that health promotion and surveillance of diseases with epidemic potential were part of their responsibilities (Fig. 1).

**Table 2:** Average indexes of the decision space of community health workers and community volunteers in the implementation of the national community health policy in Guinea, February 2022

Communes	Number	De jure index	De facto index	Capacity index	Accountability index
–	–	Mean (SD)	Mean (SD)	Mean (SD)	Mean (SD)
Fully implemented communes	41	12.00 (1.77)	22.20 (5.89)	8.66 (2.54)	3.98 (1.27)
Partially implemented communes	61	12.08 (1.66)	19.20 (6.50)	8.80 (2.58)	5.13 (1.62)
Control communes	66	11.68 (1.66)	20.59 (6.07)	7.74 (2.60)	3.65 (1.31)
Total	168	11.90 (1.70)	20.48 (6.26)	8.35 (2.61)	4.27 (1.56)
*Maximum index*	*–*	*13*	*34*	*16*	*8*
*P‑value of Bartlett*	*–*	*0.863*	*0.766*	*0.986*	*0.142*

The level of organizational and financial capacity reported by CHW and RECO was relatively low and was similar in all three types of communes (*p* = 0.986). Out of a total of 16 characteristics, the average capacity index was 8.35 (SD = 2.61; capacity index—organizational and financial capacity). Notably, only 47% of CHW/RECO owned a motorcycle as a means of transportation to carry out their activities. The distances to be covered in their assigned catchment areas are sometimes considerable, so the lack of independent transport likely hinders successful implementation of the community health policy.

**Fig. 2:**
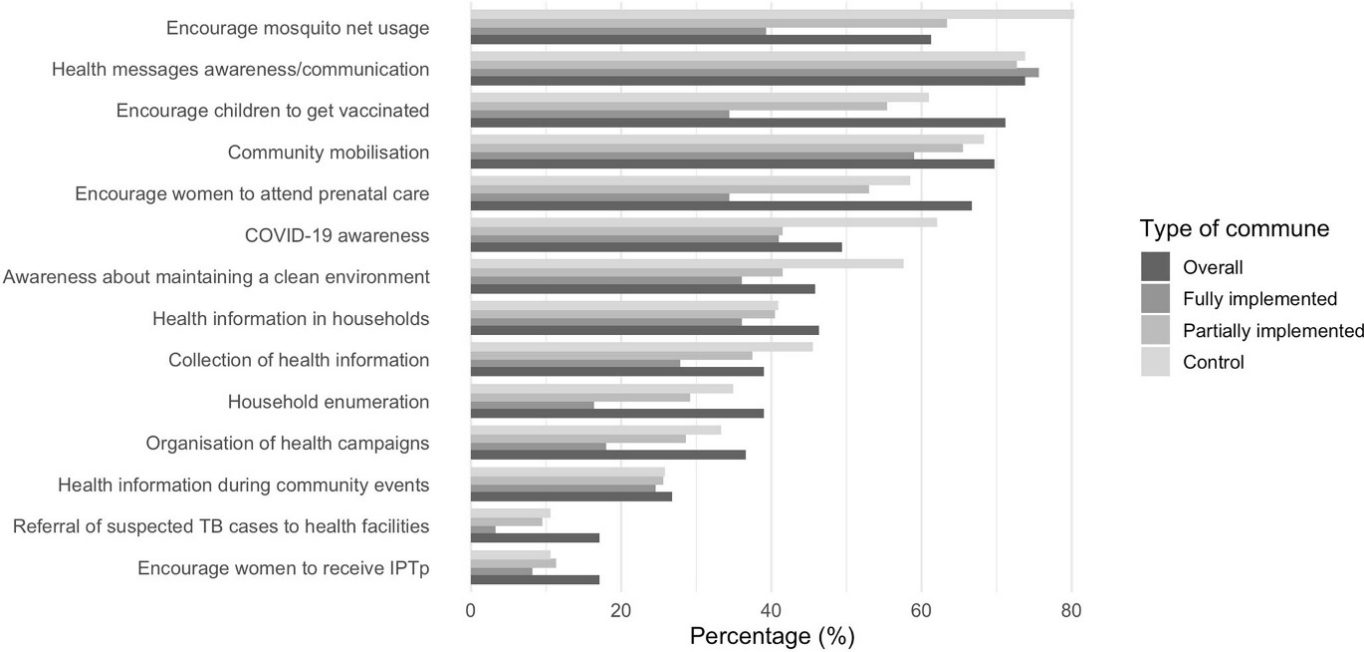
Distribution of community health workers and community volunteers according to the various preventive and curative services offered by type of commune, Guinea, February 2022. *TB* tuberculosis, *IPTp* intermittent preventive treatment in pregnancy

The level of implementation by CHW/RECO of their responsibilities was high. Out of a total of 34 community health policy activities (de facto index—implementation of assigned roles), an average of 20.48 (SD = 6.26) were implemented by CHW/RECO. That average was similar among the three types of communes (*p* = 0.766). For example, more than nine out of 10 CHW/RECO reported offering preventive or curative services (94.0%). At least 60% of them reported malaria screening, community mobilization, referrals to the health facility, and promotion of impregnated mosquito nets as preventive care in the community (Figs. 1 and 2).

One difference by commune type was the proportion of CHW/RECO who said they had participated in drawing up their health center’s annual budget plan, which was higher in fully implemented communes (29.3% [95% CI: 17.1–45.3]) than in partially implemented communes (13.1% [95% CI: 6.6–24.4]) and control communes (7.6% [95% CI: 3.1–17.2]).

### Accountability

When examining the level of accountability of health system actors across different commune types—using summative indices—there was little variation observed. On average, communes scored 4.27 (SD = 1.56) out of a total of eight items, regardless of commune type. One notable area of difference was the proportion of CHW/RECO who reported receiving sufficient resources to carry out their responsibilities during the last quarter. The average across all commune types was 33.3%, but that proportion was higher in partially implemented communes (60.7% [95% CI: 47.7–72.2]) than in fully implemented communes (19.5% [95% CI: 9.8–35.0]) and control communes (16.7% [95% CI: 9.4–27.9]). Across all communes, on average 78.0% reported that there were equal opportunity policies for women and men.

Across all commune types, there were consistently high reports of performance monitoring and mechanisms for incentives. Among RECO, 91.1% reported that their work performance had been monitored during the last quarter, largely by health center managers (57.5%), the CHW (24.8%), and the implementing nongovernmental organization partners (9.8%). Among CHW/RECO, 72.6% across all commune types reported that there was a mechanism of rewards or incentives for good performance.

Mayors often play a significant role in accountability, particularly among fully implemented communes. Specifically, 50.6% of CHW/RECO said that it was the mayors who controlled salary decisions (73.2% in fully implemented communes, compared with 50.8% in partially implemented communes and 36.4% in control communes). Additionally, in partially implemented communes, 93.3% (95% CI: 83.2–97.5) of CHW/RECO stated that it was the mayor who made decisions concerning the recruitment and/or travel of CHW/RECO (70.7% in fully implemented communes compared to 47.0% in control communes).

### Effect of commune type on decision space and routine health services

The bivariate and multivariate analysis of the effect of type of commune on decision space showed that the type of commune was not the primary predictor of CHW/RECO’s decision space in the implementation of the NCHP (Table 3 and Supplementary Tables 1–3*)*. There was no significant association between type of commune and de jure decision space (Table 3). Univariate analysis did not show an association between type of commune and the de facto decision space index (Supplementary Table 1), while multivariate analysis revealed a negative association between partially implemented communes and the de facto decision space index compared to control communes (*p* = 0.003). With regard to the commune capacity index, the results indicated that partially implemented communes were positively associated with higher capacity levels compared to control communes (Supplementary Table 2). There was no difference between control communes and fully implemented communes on capacity.

**Table 3:** Univariate and multivariate analysis of the index of the de jure decision space of community health workers and community volunteers in the implementation of the national community health policy in Guinea, February 2022

	Univariate		Multivariate	
Variables	coefficient [95% CI]	*p*-value	coefficient [95% CI]	*p*-value
*Type of commune*				
Fully implemented communes	0.318 [–0.344; 0.980]	0.344	0.331 [–0.328; 0.991]	0.323
Partially implemented communes	0.400 [–0.191; 0.991]	0.183	0.438 [–0.156; 1.032]	0.147
Control communes	Reference	–	Reference	–
*Sex*				
Female	0.222 [–0.331; 0.775]	0.430	–	–
Male	Reference	–	–	–
*Current position*				
Community health worker (CHW)	0.787 [0.269; 1305]	0.003	0.645 [0.117; 1.172]	0.017
Community volunteer	Reference	–	Reference	–
Proportion of rural population	−0.003 [–0.031; 0.024]	0.821	–	–
Proportion of population living below the national poverty line	−0.053 [–0.113; 0.008]	0.089	–	–
Target population	−0.000[–0.000; 0.000]	0.277	–	–
Number of confirmed malaria cases	0.000 [–0.000; 0.001]	0.196	–	–
Number of screened malaria cases	0.000 [–0.000; 0.001]	0.556	–	–
Coverage of assisted deliveries	−0.003 [–0.021; 0.014]	0.699	–	–
Coverage of prenatal care	0.024 [–0.108; 0.157]	0.719	–	–
BCG vaccine coverage	0.011 [0.000; 0.021]	0.041	–	–
Penta3 vaccine coverage	0.013 [0.003; 0.024]	0.014	0.014 [0.003; 0.024]	0.011
Malaria cases screened by CHW	0.001 [–0.001; 0.002]	0.313	–	–

Compared to RECO, the presence of CHW was also positively associated with increased de jure space (Table 3), de facto space (Supplementary Table 1), and increased capacity (Supplementary Table 2); *p* < 0.050. We also observed that an increase in commune population size was negatively associated with the index of de facto decision space (*p* < 0.001).

Results also show that a larger de jure space was associated with higher BCG vaccination coverage (*p* = 0.041) in univariate analysis and higher penta 3 vaccine coverage in bivariate (*p* = 0.014) and multivariate (*p* = 0.011) analysis. Likewise, the increase in the number of malaria cases detected by CHW/RECO was associated with both a higher de facto decision space and higher accountability (Supplementary Table 3).

## Discussion

The aim of this study was to assess the extent to which CHW/RECO are aware of their roles and responsibilities in the context of the national community health policy and their capacity to implement them. The study hypothesized that fully implemented communes would have the highest decision-making space, followed by partially implementing communes and control communes. However, the results showed that commune type was not the primary determinant of decision-making space. This suggests that factors other than implementation level, such as vaccination coverage, may play a more significant role. This emphasizes the need to focus on strengthening CHW/RECO roles and ensuring that adequate resources are available to support their work rather than solely relying on implementation status.

The results of the study revealed that CHW/RECO had limited knowledge of the NCHP and decentralization policies and strategies. Moreover, levels of knowledge were similar across all types of communes. Considering broader contextual factors that influence decision-making spaces such as knowledge of policy and strategy documents by CHW/RECO is, therefore, important.

The CHW/RECO, who implement the minimum package of community health activities, had higher levels of knowledge of community health activities and higher involvement (greater de facto decision space) than commune-level actors or central-level and regional-level actors. The encouraging results observed among CHW/RECO in terms of their knowledge and implementation could be explained by the contents of their training program, which includes activities linked to the community health policy and decentralization. However, it is important to note that even though CHW/RECO reported a high level of involvement, this did not necessarily translate into consistent implementation of community health activities in the field. Consistent with these findings, an evaluation of the Health Service Delivery Activity in Guinea in 2020 indicated that CHW were providing inconsistent and variable services depending on the regions of implementation [27]. Those gaps between theory and practice highlight several challenges to the effective implementation of community health responsibilities for all levels, namely, issues related to implementation fidelity. Key challenges identified include inadequate, inconsistent, and fragmented financing; delayed payments; and a shortage of human resources, all of which hinder the successful implementation of the NCHP. These challenges are consistent with the literature on the fragmented nature of community health programs across other country settings [28] and the need for more coordinated and sustainable funding mechanisms [29]. Despite these challenges, over half of respondents reported that the NCHP was sustainable, a promising indication.

Another key challenge is the shortage of human resources, both in terms of number and quality, particularly in relation to adequate training. For example, when the number of CHW/RECO falls below the recommended ratio relative to the population they serve, their ability to deliver effective care is compromised. Additionally, in some areas, RECO are responsible for covering vast catchment zones, which further limits their capacity to provide comprehensive and timely services. This challenge is compounded by staffing shortages in certain health centers, which in turn affects the support and coordination needed for CHW and RECO to perform their roles effectively.

Developing and implementing a capacity-strengthening plan for CHW/RECO, adapted to their specific roles and responsibilities, could help improve their performance and related health indicators. This plan could include recruiting additional CHW/RECO, as well as strengthening the capacity of existing CHW/RECO, to improve the coverage of certain health services where gaps exist, such as reaching zero-dose or underimmunized children. Ensuring more consistent supportive supervision could also be a means to provide continuous capacity-strengthening for CHW and RECO.

Strengthening the accountability of CHW and RECO, and maintaining it over time, was also identified as a need. The results indicated that the accountability of CHW/RECO was relatively higher in partially implemented communes that had just begun implementing the NCHP than in the other two types of communes. To ensure greater accountability of CHW/RECO and other implementers toward community members rather than to partners and donors, it is necessary to set up or strengthen mechanisms for funding salaries and stipends through local government. At a workshop held to validate the results of this study to stakeholders in Guinea, a recommendation was made that the federal government should guarantee that CHW/RECO, who are the main implementing actors, should be paid, to strengthen their accountability to the community. That payment should also involve the community itself so that it can exercise greater oversight over CHW/RECO. To date, a law has been passed to that effect, creating a local government position that guarantees payment of CHW/RECO [30]. This change in payment mechanism will hopefully strengthen both accountability of CHW/RECO to their communities and sustainability.

This study has several limitations that should be considered in the overall interpretation of its results. The decision space survey used self-reported, cross-sectional survey data from participants at various levels of the health system in Guinea, and these self-reported data could be influenced by social desirability bias. To mitigate social desirability bias, we tried to ask specific, easily understandable questions geared toward what the actors were doing, rather than what they wanted or thought they should be doing. In addition, we trained and supervised field collection teams to, for example, avoid asking leading questions and pay attention to body language, facial expressions, etc., to minimize social desirability bias on the part of respondents.

This study used OLS regression to analyze and model the relationship between decision space and certain covariates; OLS regression is potentially subject to confounding and omitting variable bias. Using theory and literature to select controls for regression analyses may have helped to reduce, but not eliminate, the threat of omitted variable bias. The results from the regression analyses indicate that contrary to our hypotheses, commune type (fully implementing, partially implementing, or control) was not the main predictor of CHW, and proximity could have had a spillover effect in control communes. Moreover, we also believe that the lack of difference between fully implemented communes and other communes is because, at the time of data collection, the fully implemented communes were not receiving financial support for paying CHW/RECO, thereby influencing their performance. Further, new policies such as the NCHP often produce initial improvements that decline over time as focus, attention, and support decrease and other priority activities are introduced.

The results of this study might not be generalizable to regions of Guinea not included in the study. Further, this is a descriptive, observational study. Therefore, we are unable to draw causal conclusions regarding the study results, so the results of this study should be treated as associational and not causal.

Beyond these limitations, this study provides novel evidence of the importance of CHW/RECO in the implementation of the NCHP in Guinea. Its findings can help to inform future community health evaluations in Guinea and other countries implementing community health programs, as well as other researchers exploring decision space in the context of decentralized health systems.

## Conclusion

This study underscores the pivotal role of CHW/RECO in implementing Guinea‘s NCHP. It shows high levels of knowledge and involvement among CHW and RECO. However, challenges such as resource limitations and inconsistent implementation persist. Therefore, there is a need for enhanced capacity-building and sustainable funding mechanisms to improve accountability and service delivery of community actors. Such investment will strengthen the decision space and performance of decentralized health systems and result in better health outcomes.

## Supplementary Information

Univariate and multivariate analysis of the index of the de facto decision space, the index of the capacity and the accountability index of CHW and RECO in the implementation of the NCHP in Guinea, February 2022.

## Data Availability

All relevant data are within the paper and its supporting information files. The raw data supporting the conclusions of this article will be made available by the authors upon request.

## Declarations

All procedures performed in studies involving human participants or on human tissue were in accordance with the ethical standards of the institutional and/or national research committee and with the 1975 Helsinki declaration and its later amendments or comparable ethical standards. The study protocol was approved by the Guinea National Ethics Committee for Health Research in Guinea (No. 161/CNERS/2021) and the Harvard T.H. Chan School of Public Health (IRB21-1162). Informed consent was obtained from all individual participants included in the study.

## References

[1] Programme des Nations Unies pour le VIH/SIDA (2015) 2 millions d’agents de santé communautaires en Afrique—Tirer pleinement profit du dividende démographique, mettre fin à l’épidémie de sida et assurer durablement la santé pour tous en Afrique. https://www.unaids.org/sites/default/files/media_asset/African2mCHW_fr.pdf. Accessed 13 Jan 2025

[2] Le Roux KW, Almirol E, Rezvan PH et al (2020) Community health workers impact on maternal and child health outcomes in rural south africa—a non-randomized two-group comparison study. Bmc Public Health 20:1–14. 10.1186/S12889-020-09468-W/FIGURES/3 32943043 10.1186/s12889-020-09468-w PMC7496216

[3] Schneider H, Okello D, Lehmann U (2016) The global pendulum swing towards community health workers in low- and middle-income countries: a scoping review of trends, geographical distribution and programmatic orientations, 2005 to 2014. Hum Resour Health. 10.1186/S12960-016-0163-2 27784298 10.1186/s12960-016-0163-2 PMC5081930

[4] Gilmore B, McAuliffe E (2013) Effectiveness of community health workers delivering preventive interventions for maternal and child health in low- and middle-income countries: A systematic review. BMC Public Health 13:1–14. 10.1186/1471-2458-13-847/TABLES/2 24034792 10.1186/1471-2458-13-847 PMC3848754

[5] Olaniran A, Smith H, Unkels R et al (2017) Who is a community health worker?—a systematic review of definitions. Glob Health Action. 10.1080/16549716.2017.1272223 28222653 10.1080/16549716.2017.1272223 PMC5328349

[6] Alhassan JAK, Wills O (2024) Public health surveillance through community health workers: a scoping review of evidence from 25 low-income and middle-income countries. BMJ Open 14:e79776. 10.1136/BMJOPEN-2023-079776 38582533 10.1136/bmjopen-2023-079776 PMC11002386

[7] World Health Organization (2021) What do we know about community health workers? A systematic review of existing reviews. https://www.who.int/publications/i/item/what-do-we-know-about-community-health-workers-a-systematic-review-of-existing-reviews. Accessed 2 Apr 2025

[8] Masis L, Gichaga A, Zerayacob T et al (2021) Community health workers at the dawn of a new era: 4. Programme financing. Health Res Policy Syst 19:1–17. 10.1186/S12961-021-00751-9/TABLES/6 34641893 10.1186/s12961-021-00751-9 PMC8506106

[9] Manor J (1999) The political economy of democratic decentralization, p 133

[10] Odii A, Etiaba E, Onwujekwe O (2024) Examining the roles and relationships of actors in community health systems in Nigeria through the lens of the expanded health systems framework. BMJ Glob Health 9:14610. 10.1136/BMJGH-2023-014610 10.1136/bmjgh-2023-014610 PMC11499808 39433401

[11] Masaba BB, Moturi JK, Taiswa J, Mmusi-Phetoe RM (2020) Devolution of healthcare system in Kenya: progress and challenges. Public Health 189:135–140. 10.1016/J.PUHE.2020.10.001 33227596 10.1016/j.puhe.2020.10.001

[12] McCollum R, Limato R, Otiso L et al (2018) Health system governance following devolution: comparing experiences of decentralisation in Kenya and Indonesia. Bmj Glob Health 3:e939. 10.1136/BMJGH-2018-000939 30294460 10.1136/bmjgh-2018-000939 PMC6169670

[13] Sapkota S, Dhakal A, Rushton S et al (2023) The impact of decentralisation on health systems: a systematic review of reviews. BMJ Glob Health 8:13317. 10.1136/BMJGH-2023-013317 10.1136/bmjgh-2023-013317 PMC10749071 38135299

[14] Kapuya HA, Maluka SO, Hurtig AK, Sebastian MS (2024) Assessing community awareness and participation in health facility governing committees in two districts of Tanzania: a cross-sectional study. Arch Public Health 82:1–8. 10.1186/S13690-024-01415-0/TABLES/3 39472995 10.1186/s13690-024-01415-0 PMC11520387

[15] Schaaf M, Fox J, Topp SM et al (2018) Community health workers and accountability: reflections from an international “think-in. Int J Equity Health 17:1–5. 10.1186/S12939-018-0781-5/METRICS 29801493 10.1186/s12939-018-0781-5 PMC5970525

[16] Kruk ME, Gage AD, Arsenault C et al (2018) High-quality health systems in the sustainable development goals era: time for a revolution. Lancet Glob Health 6:e1196. 10.1016/S2214-109X(18)30386-3 30196093 10.1016/S2214-109X(18)30386-3 PMC7734391

[17] Ministère de la Santé de Guinée (2017) Politique Nationale de Santé Communautaire. https://cnls-guineeconakry.org/wp-content/uploads/2019/09/document_de_politique_sante_communautaire.pdf. Accessed 13 Jan 2025

[18] (2025) UNICEF Le Programme National d’Appui aux Communes de Convergence. https://www.unicef.org/guinea/le-programme-national-dappui-aux-communes-de-convergence. Accessed 2013

[19] Bossert T (1998) Analyzing the decentralization of health systems in developing countries: decision space, innovation and performance. Soc Sci Med 47:1513–1527. 10.1016/S0277-9536(98)00234-2 9823047 10.1016/s0277-9536(98)00234-2

[20] orld Bank Group Literacy rate, adult total (% of people ages 15 and above)—Least developed countries: UN classification, Guinea. https://data.worldbank.org/indicator/SE.ADT.LITR.ZS?locations=XL-GN. Accessed 18 Nov 2024

[21] Institut National de statistique|Guinea (2023) Annuaire statistique 2022. https://www.stat-guinee.org/index.php/publications-ins/89-publications-annuelles. Accessed 16 Sept 2023

[22] Bossert TJ, Mitchell AD (2011) Health sector decentralization and local decision-making: Decision space, institutional capacities and accountability in Pakistan. Soc Sci Med 72:39–48. 10.1016/J.SOCSCIMED.2010.10.019 21134705 10.1016/j.socscimed.2010.10.019

[23] Delamou A, Grovogui FM, Miller L et al (2023) Implementation research protocol on the national community health policy in Guinea: a sequential mixed-methods study using a decision space approach. PLoS ONE. 10.1371/JOURNAL.PONE.0280651 36662762 10.1371/journal.pone.0280651 PMC9858093

[24] Marchal B, van Belle S, van Olmen J et al (2012) Is realist evaluation keeping its promise? A review of published empirical studies in the field of health. Syst Res 18:192–212. 10.1177/1356389012442444

[25] KoboToolbox (2025) Powerful and intuitive data collection tools to make an impact. https://www.kobotoolbox.org. Accessed 31 Mar 2025

[26] Roman TE, Cleary S, McIntyre D (2017) Exploring the functioning of decision space: a review of the available health systems literature. Int J Health Policy Manag 6:365. 10.15171/IJHPM.2017.26 28812832 10.15171/ijhpm.2017.26 PMC5505106

[27] El Ayadi A, Hilber MA, Delamou A et al (2020) Final evaluation of the USAID Guinea health service delivery activity: integrated health service delivery in the post-Ebola context. https://iscollab.org/wp-content/uploads/Guinea-HSD-Full-Report_english_complete.pdf. Accessed 13 Jan 2025

[28] Tulenko K, Møgedal S, Afzal MM et al (2013) Community health workers for universal health-care coverage: from fragmentation to synergy. Bull World Health Organ 91:847–852. 10.2471/BLT.13.118745 24347709 10.2471/BLT.13.118745 PMC3853952

[29] Liberia Ministry of Health (2023) The Monrovia Call to action launched by the Liberia ministry of health at 2023 CHW symposium—3rd international community health worker symposium. https://chwsymposiumliberia2023.org/2023/03/27/the-monrovia-call-to-action-launched-by-the-liberia-ministry-of-health-at-2023-chw-symposium/. Accessed 13 Jan 2025

[30] Health systems strengthening accelerator new law mandates salaries for Guinea’s community health workers. https://www.acceleratehss.org/2023/04/03/new-law-mandates-salaries-for-guineas-community-health-workers/. Accessed 13 Jan 2025

