# Supplementary material for: Implementation of the national community health policy in Guinea: a decision space analysis of the roles and responsibilities of community health workers
Source: Bundesgesundheitsblatt Gesundheitsforschung Gesundheitsschutz. 2025 Jun 20;68(7):738–46. doi: 10.1007/s00103-025-04076-8 (PMC12254089; doi:10.1007/s00103-025-04076-8)
Supplement: Supplementary file 1 — Univariate and multivariate analysis of the index of the de facto decision space, the index of the capacity and the accountability index of CHW and RECO in the implementation of the NCHP in Guinea, February 2022. [file 103_2025_4076_MOESM1_ESM.pdf]

Supplementary materials

Supplementary Table 1: Univariate and multivariate analysis of the index of the *de facto* decision space of CHW and RECO in the implementation of the NCHP in Guinea, February 2022

	Univariate		Multivariate	
**Variables**	**Coef. [95%CI]**	**p-value**	**Coef. [95%CI]**	**p-value**
Type of commune				
Fully implemented communes	1.604[-0.825; 4.034]	0.194	0.768[-1.516; 3.052]	0.508
Partially implemented communes	-1.394[-3.564; 0.776]	0.206	-3.384[-5.606; -1.162]	0.003
Control communes	Ref.		Ref.	
Sex				
Female	-1.330[-3.377; 0.718]	0.202		
Male	Ref.			
Current position				
CHW (Community Health Worker)	2.540[0.603; 4.476]	0.010	2.425[0.598; 4.253]	0.010
RECO (Community volunteers)	Ref.		Ref.	
Proportion of rural population	-0.186[-0.284; -0.089]	<0.001	-0.211[-0.315; -0.108]	<0.001
Proportion of population living below the national poverty line	0.231[0.007; 0.456]	0.044		
Target population	-0.000[-0.000; 0.000]	0.374	-0.000[-0.000; -0.000]	0.046
Number of confirmed malaria cases	0.002[-0.000; 0.005]	0.069	0.000[-0.005; 0.005]	0.895
Number of screened malaria cases	0.002[-0.000; 0.004]	0.053		
Coverage of assisted deliveries	-0.057[-0.122; 0.008]	0.085		
Coverage of prenatal care	0.068[-0.423; 0.560]	0.785		
BCG vaccine coverage	-0.016[-0.054; 0.023]	0.425		
Penta3 vaccine coverage	0.029[-0.011; 0.068]	0.153		
Malaria cases screened by CHW	0.006[0.001; 0.011]	0.022	0.006[-0.000; 0.013]	0.059

Supplementary Table 2: Univariate and multivariate analysis of the index of the capacity of CHW and RECO in the implementation of the NCHP in Guinea, February 2022

	Univariate		Multivariate	
**Variables**	**Coef.[95%CI]**	**p-value**	**Coef.[95%CI]**	**p-value**
Type of commune				
Fully implemented communes	0.916[-0.096; 1928]	0.076	0.357[-0.523; 1.236]	0.424
Partially implemented communes	1.061[0.157; 1.964]	0.022	1.475[0.620; 2.330]	0.001
Control communes	Ref.		Ref.	
Sex				
Female	-0.044[-0.902; 0.813]	0.919		
Male	Ref.			
Current position				
CHW (Community Health Worker)	2.359[1.619; 3.098]	<0.001	2.509[1.794; 3.224]	<0.001
RECO (Community Volunteer)	Ref.		Ref.	
Proportion of rural population	0.019[-0.023; 0.061]	0.380		
Proportion of population living below the national poverty line	-0.078[-0.172; 0.016]	0.104		
Target population	0.000[-0.000; 0.000]	0.136		
Number of confirmed malaria cases	0.000[-0.001; 0.001]	0.996		
Number of screened malaria cases	-0.000[-0.001; 0.001]	0.947		
Coverage of assisted deliveries	0.015[-0.012; 0.042]	0.276		
Coverage of prenatal care	-0.044[-0.249; 0.160]	0.669		
BCG vaccine coverage	-0.016[-0.032; 0.000]	0.055		
Penta3 vaccine coverage	-0.003[-0.020; 0.013]	0.697		
Malaria cases screened by CHW	-0.001[-0.003; 0.001]	0.259		

Supplementary Table 3: Univariate and multivariate analysis of the accountability index of CHW and RECO in the implementation of the NCHP in Guinea, February 2022

	Univariate		Multivariate	
**Variables**	**Coef.[95%CI]**	**p-value**	**Coef.[95%CI]**	**p-value**
Type of commune				
Fully implemented communes	0.324[-0.234; 0.882]	0.253	0.367[-0.185; 0.918]	0.191
Partially implemented communes	1.480[0.982; 1.978]	<0.001	1.537[1.043; 2.031]	<0.001
Control communes	Ref.		Ref.	
Sex				
Female	-0.061[-0.574; 0.453]	0.816		
Male	Ref.			
Current position				
CHW (Community Health Worker)	0.384[-0.105; 0.873]	0.123		
RECO (Community volunteers)	Ref.			
Proportion of rural population	-0.016[-0.042; 0.009]	0.205		
Proportion of population living below the national poverty line	-0.040[-0.097; 0.016]	0.161		
Target population	-0.000[-0.000; 0.000]	0.493		
Number of confirmed malaria cases	0.001[-0.000; 0.001]	0.110	0.001[0.000; 0.001]	0.020
Number of screened malaria cases	0.000[-0.000; 0.001]	0.316		
Coverage of assisted deliveries	0.019[0.003; 0.035]	0.023		
Coverage of prenatal care	0.119[-0.003; 0.240]	0.055		
BCG vaccine coverage	0.007[-0.003; 0.016]	0.177		
Penta3 vaccine coverage	-0.005[-0.014; 0.005]	0.368		
Malaria cases screened by CHW	0.002[0.001; 0.003]	0.002		
